# Reproduction and Population Dynamics as Biotypic Markers of Russian Wheat Aphid *Diuraphis noxia* (Kurdjumov)

**DOI:** 10.3390/insects7020012

**Published:** 2016-04-02

**Authors:** Watson Ngenya, Joyce Malinga, Isaiah Tabu, Emily Masinde

**Affiliations:** 1 Department of Crops, Horticulture and Soils, Egerton University, Egerton 536-20115, Kenya; immtabu@yahoo.com (I.T.); masinde.emily11@gmail.com (E.M.); 2 Kenya Agricultural and Livestock Research Organization, Food crops institute, Kitale 450-30200, Kenya; joycemalinga@kalro.org

**Keywords:** *Diuraphis noxia*, population, preference, reproduction

## Abstract

Russian wheat aphid *Diuraphis noxia* (Kurdjumov) is widely established in wheat-growing countries where it causes significant economic losses. The development and use of Russian wheat aphid (RWA)-resistant wheat varieties has been constrained by the variation in resident RWA populations and the evolution of virulent biotypes. An experiment was set up at the Kenya Agricultural and Livestock Research Organization (KALRO), Njoro, to characterize RWA populations based on phenotypic characteristics of reproduction, development and population dynamics. RWA populations from the regions of Eldoret, Mau Narok and Njoro were used in the study. A factorial experiment was set up in randomized complete block design replicated eleven times. A single day-old nymph was placed on a new, fully-open leaf in a 0.5 cm-diameter clear plastic straw leaf cage and observed daily for its entire lifetime. The results showed that there were variations in aphid lifespan, reproductive longevity and aphid fecundity between populations, indicating that the phenotypic markers used to determine biotypes were good enough to show distinct biotypes among populations of the RWA in Kenya. Further, the study concluded that the use of phenotypic life and reproductive markers was a valid way of characterizing biotypes of RWA worldwide.

## 1. Introduction

Russian wheat aphid (RWA), *Diuraphis noxia* (Kurdjumov), is a major pest of wheat and barley, especially in warm climates, causing massive grain losses in severe infestation. Losses of up to 90% of the crop have been reported in severe infestations in Kenya, the USA, South Africa and Iran [1,2,3]. Traditionally, long-term sustainable management of RWA relies on host plant resistance (HPR). Chemical control reduces environmental quality and kills biocontrol agents, thereby reducing their efficacy [4,5,6]. The use of HPR is one of the least expensive and most important ways of protecting the crop from losses attributed to RWA damage and, at the same time, protecting the environment from pesticide contamination [7]. Resistance to RWA is genetically controlled and is expressed as antibiosis, non-preference (antixenosis) and tolerance. HPR is an integral component of integrated pest management (IPM), since it can be combined with host plant resistance and cultural control and even chemical control to achieve sustainable management of the pest population below the economic threshold. IPM is recommended as the most appropriate and desirable pest control strategy for RWA management [8,9].

Host plant resistance, the all-important RWA management strategy, is not effective in some instances due to the evolution of RWA biotypes [10,11,12,13]. These are variants of the pest that are able to overcome host plant resistance mechanisms, especially antibiosis and antixenosis [14]. Nine RWA resistance genes, *Dn1*, *Dn2*, *Dn3*, *Dn4*, *Dn5*, *Dn6*, *Dn7*, *Dn8* and *Dn9*, for wheat have been characterized by various researchers, mainly in South Africa and the USA. The recessive *Dn3* gene originated from *T. tauschii*, whereas the dominant gene *Dn7* resulted from an inter-generic transfer from rye to wheat [15]. Biotypes of RWA started to be noticed in 1989 when RWAs in Syria and Kyrgiz were found to be virulent to the *Dn4* resistance gene in wheat [10,13]. Haley *et al.* [11] identified a virulent biotype in Colorado that could acutely damage wheat with any one of the eight of the nine *Diuraphis noxia* (*Dn*) resistance genes, with the exception of *Dn7*, and designated this biotype as Biotype 2. The other Russian wheat aphid biotypes, all characterized in the USA, Biotype 3, Biotype 4 and Biotype 5, have the ability to differentially damage wheat with *Dn1* to *Dn9* resistance genes in wheat [16,17], whereas RWASA1 and RWASA2 have been characterized in South Africa [18]. RWA biotypes generally show phenotypic differences in terms of reproduction, population increase and differential virulence on different resistant host genotypes [11,13,18,19]. Reproductive decisions are based on plant cues and cues based on reproductive decisions, which may be an additional determinant of fitness [20] and, thus, a basis for biotyping RWA populations. Female insects maximize species fitness by ovipositing on high quality hosts [21]. The ability to locate a suitable host for colonization and a corresponding superior reproductive ability and survival are outstanding features for ecologically-successful pests. Ecologically-successful biotypes would have shorter generation times, be prolific and live longer on a host, making these phenotypic markers possible effective indicators of biotypes. The objectives of the study are to understand if these phenotypic markers could be used for characterizing new biotypes. An experiment was set up to characterize RWA biotypes in Kenya based on the phenotypic markers of reproduction, growth and survival on selected wheat genotypes as hosts.

## 2. Experimental Section

### 2.1. Site Description

This research was conducted at Kenya Agricultural Research Institute, National Plant Breeding Station, Njoro. It is located in the lower highlands (LH_3_), standing at an altitude of 2166 meters above sea level [22].

### 2.2. Aphid Collection

A single Russian wheat aphid was collected from symptomatic bread wheat in January 2012 at three different wheat growing regions that contribute significantly to the total annual wheat harvest in Kenya (Table 1). The aphids were identified according to descriptions given by [23] and taken to the Kenya Agricultural and Livestock Research organization (KALRO), Njoro, research station, where the experiments were conducted.

### 2.3. Aphid Rearing

The rearing plants (susceptible wheat variety Kenya Pasa) were planted in a sterilized mixture of forest soil to manure ratio of 3:1. The potting mixture was amended with di-ammonium phosphate at the rate of 50 kg/Ha. Three seeds were planted in a 1-L plastic pot and the emergent seedlings caged with ventilated polyester mesh (0.5 mm in diameter) to keep the seedlings clean from aphid contamination in the greenhouse. The plants were watered regularly by setting plant pots in a water bath for an hour after every two days so that the seedlings were not water stressed.

A single adult female RWA was settled in the leaf whorl of clean susceptible wheat seedlings of variety Kenya Pasa (Pasa) at Growth Stage 12 [24] using a fine hair brush. The inoculated seedlings were caged using ventilated polyester mesh on wire support and kept in ventilated glass insect rearing boxes in the greenhouse at 18 ± 2 °C under natural light conditions. The aphids were allowed to multiply freely to form a colony. The insect rearing boxes were designated for specific populations and were kept a minimum of 10 m from each other to eliminate mixing of the populations. The aphid colony in an insect rearing box was named after the wheat growing area that the founder aphid was collected from and designated as either the Njoro population, the Eldoret population or the Mau Narok population.

### 2.4. Host Genotypes

The wheat genotypes Kenya Kwale (Kwale) and KRWA9 were used in the study and were sourced from Kalro, Njoro. Kwale, released in 1975, is a popular Kenyan variety grown in most wheat growing areas. It is however susceptible to RWA and was thus used as the susceptible control. KRWA9, on the other hand, is an introduced line identified for resistance to RWA and is currently used in the RWA resistance breeding program in Kenya.

### 2.5. Growth of RWA Populations on Selected Wheat Genotypes

The experiment was a two-factor experiment in a completely randomized design with eleven replications. The two factors were aphid population and wheat genotype. Aphid biotype had three levels, Eldoret, Mau Narok and Njoro, while two wheat genotypes, Kenya Kwale (Kwale) and KRWA9, were used in the study.

Two seeds of the Kwale and KRWA9 genotypes were planted in individual pots filled with a sterilized mixture of forest soil to manure ratio of 3:1 and supplemented with DAP. Thirty three pots of Kwale and thirty three pots of KRWA9 were planted and arranged in a randomized complete block design on a greenhouse bench. After germination, the seedlings were thinned to leave one seedling per pot. When the test plants reached the two-leaf stage, the midsection of one leaf was enclosed inside a leaf cage made from a clear plastic drinking straw measuring 0.5 cm in diameter and 3 cm in length. The straw leaf cage had previously been ventilated with 20 holes made by piercing the straw section with an insect pin. The leaf tip was placed at one end of the cage and the cage moved towards the plant stem until the middle of the leaf was enclosed. Each covered plant leaf section was then infested with one adult RWA from a RWA biotype and both ends of the cage plugged with a piece of cotton wool. After 12 h, the cages were unplugged and the adult and the born nymphs removed to leave only one nymph per cage. The nymphs retained and caged on leaves of the test plants were the same age with at most a 12-h variation in their age. The retained nymphs were observed on a daily basis for the molting, reproduction and death of aphids. Aphids were moved to the highest fully-open fresh leaves when the caged leaf section deteriorated. The temperature for the entire duration of the experiment was 18 ± 2 °C, and the relative humidity inside the greenhouse was 65%. Test plants were watered regularly throughout the duration of the study to keep plants healthy.

Instar development time (days taken for a nymph to molt), development time (days from birth to the date of the first larviposition), reproductive longevity (time taken from the day of first reproduction to cessation of reproduction or death, whichever occurred first), total longevity (time from birth to death) and fecundity (total number of nymphs born of a single aphid during its life) were recorded. In order to determine how the host genotype influences a biotype’s rate of population increase, the intrinsic rate of population increase (*r*_m_) and cohort generation time (T_c_) for each aphid population on each wheat genotype were calculated using the method of [25].

(1) $$r_{m}=\frac{0.738(\ln Md)}{d}$$

*d* is the development time from birth to first reproduction. Effective fecundity, *M_d_*, is the number of offspring that were produced in a time *d*.

The cohort generation time (T_c_) for each population was calculated using the formula:

(2) $$T_{C}=\frac{4d}{3}$$

*d* is the development time from birth to first reproduction.

### 2.6. Data Analysis

An analysis of variance using Genstat [26] was done, and significant differences in treatment means were separated using Tukey’s HSD test at the α = 0.05 level of significance.

## 3. Results and Discussion

### Growth of RWA on Selected Wheat Genotypes

**Figure 1:**
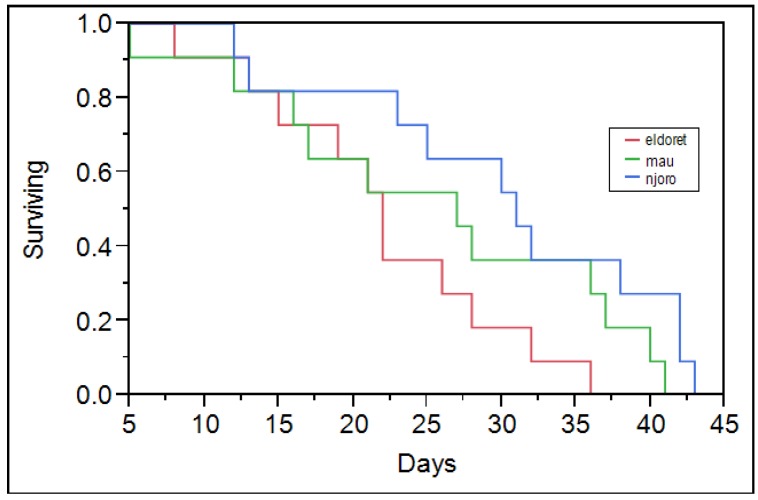
Survivorship curves for Eldoret and Mau Narok and Njoro RWA populations on KRWA9.

**Figure 2:**
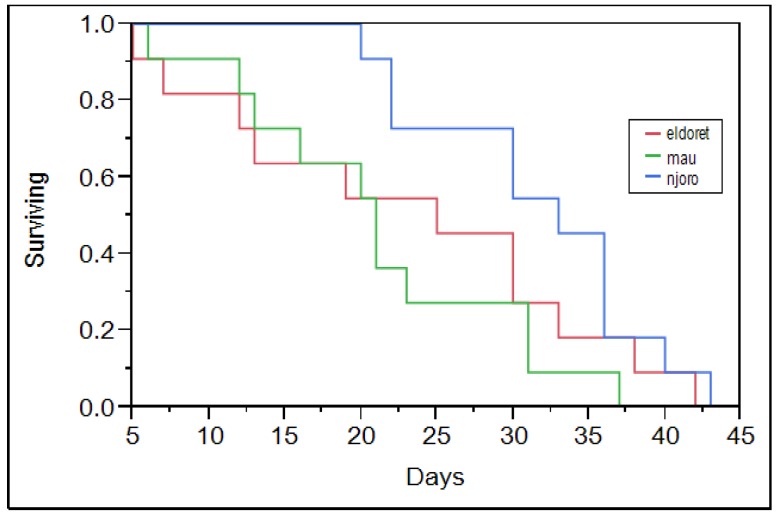
Survivorship curves for Eldoret and Mau Narok and Njoro RWA populations on Kwale.

The RWA populations did not significantly differ in their survivorship on KRWA9 and Kwale (Table 2). Eldoret and Mau Narok populations had a characteristic survivorship curve with constant mortality across all ages (Figure 1 and Figure 2). The Njoro population however had high mortality towards its maximum lifetime. Maling’a *et al* [27] noted a similar mortality trend for RWA population collected from the Njoro population. The Njoro population had low mortality among its young compared to the Eldoret and Mau Narok populations (Figure 1 and Figure 2). The difference is probably because the different populations are different. The prevailing greenhouse temperature was common to all of the RWA populations that were studied. The Njoro population, which has lived under similar conditions for a longer duration, was more adapted compared to other populations that were acclimatized in the greenhouse for three months prior to the start of the study. Njoro has a lower altitude compared to both Mau Narok and Eldoret (Table 2). Michels and Behle [28] in their study of RWA populations in Ethiopia found that the mortality of RWA increased with the increase in temperature. This could have caused the increased mortality of the Mau Narok and Eldoret populations since they were collected from regions with lower temperatures and acclimatized in the greenhouse for three months.

The aphid populations did not differ in their development time (the time from birth to first reproduction). Wheat genotypes however had a significant effect on the development time of populations of RWA (Table 3). Primary and secondary metabolites found in plant phloem exudates influence aphid development and reproduction [29,30]. Resistant plants contain higher levels of antibiotic secondary metabolites, such as hydroxamic acids, that reduce insect attack [31]. Kwale did not have RWA resistance and was more suitable for aphid development compared to KRWA9. Aphid populations took a significantly shorter time to develop on Kwale compared to KRWA9.

**Figure 3:**
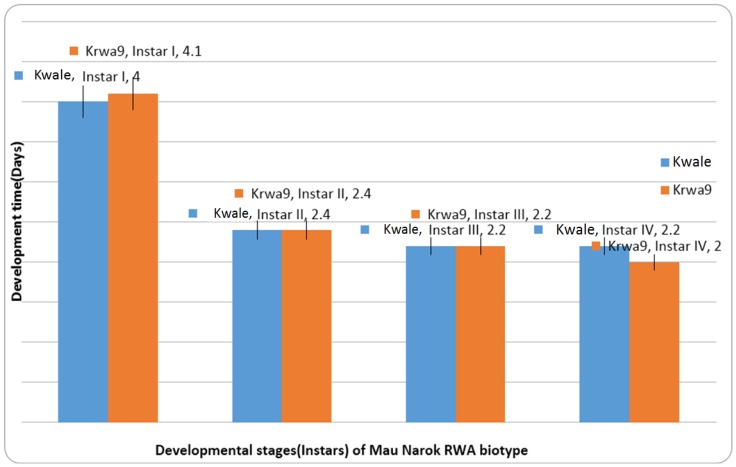
Development time of instars of the Mau Narok RWA population on Kwale and KRWA9.

There was significant host-aphid population interaction on the development time of the first instar of RWAs (Table 4). The development time of the first instar of the Mau Narok and Njoro populations was significantly different from the Eldoret population. The development time of the second instar of the Mau Narok RWA population was significantly longer compared to the Eldoret and Njoro RWA populations, which had similar development time on Kwale. Overall, the development differed significantly among RWA populations on Kwale. The Eldoret RWA population had the shortest development time on Kwale compared to Mau Narok and Njoro populations, whose development time was similar on Kwale. There were no differences in the development time of RWA populations on KRWA9 (Table 5). Second and third instars of the Mau Narok population were observed to be indifferent to the effect of variety on development time (Figure 3).This result indicates that the Mau Narok population is virulent, since the resistance of KRWA9 does not affect the development time of its instars (Table 4). There was no host by aphid population interaction on the development time of the third and fourth instars of all tested RWA populations (Table 4). The results observed are consistent with the findings by Aalbersberg *et al.* [32], Kazemi *et al.* [33] and Maling’a *et al.* [19].

Wheat genotypes did not significantly affect the reproduction time and lifespan of aphids. Host genotype, however, significantly affected the M_d_ (fecundity in a time equivalent to the aphid development time, M_d_) and total aphid fecundity. Genotype Kwale was the most suitable host, as shown by the high number of progeny produced on it (Table 5). Qing-Nian *et al.* [34] similarly noted that resistant wheat genotypes significantly decreased the population growth of the grain aphid *Sitobion avenae* and therefore noted that the oviposition behavior of herbivorous insects responds to host quality and availability [20,35,36]. All populations had significantly higher total fecundity and M_d_ (Fecundity at a time equivalent to the development time) on Kwale, a susceptible host compared to resistant genotype KRWA9. This indicates that KRWA9 has antibiosis as the mode of resistance, since it affects aphid biology. The high increase in population during a time equivalent to the development time has been cited as critical to determining the individual contribution to the population of an aphid species, because aphids contribute almost 90% of the progeny to population during this period [25].

There were significant differences in aphid reproductive time, total aphid lifespan, M_d_ (fecundity in a time equivalent to the aphid development time) and total aphid fecundity between aphid populations (Table 6). The Njoro population produced a significantly higher number of progeny and had the longest reproduction time compared to the Mau Narok and Eldoret populations. The Njoro population had a higher reproductive longevity and fecundity than the Eldoret and Mau Narok populations (Table 6). The Njoro and Eldoret populations had a higher number of progeny at a duration equivalent to the development time (M_d_), compared to Mau Narok. The differences in total aphid fecundity can be attributed to differences in aphid populations, wheat genotypes and the longevity of the reproductive time.

There was no host by population interaction on reproduction time, total lifespan, daily fecundity and fecundity of a time equivalent to the development time of RWA. However, there was a host by population interaction on total fecundity. The Eldoret and Njoro populations had the highest fecundity on Kwale, while the Njoro and Mau Narok populations had the highest progeny on KRWA9. The Eldoret population, however, had the lowest progeny on KRWA9, indicating that this genotype may be effective in managing the population buildup of the Eldoret population of RWA (Table 6). Diehl and Bush [14] defined conspecific, sympatric populations, which differ in some biological traits, as biotypes. Longevity of reproductive time, aphid lifespan and total aphid fecundity of aphid populations clearly show the Njoro population to be a distinct biotype of RWA in Kenya.

All aphid populations had a positive intrinsic rate of population increase, indicating their ability to build up populations. There was a significant effect of wheat genotype and aphid population on the intrinsic rate of the natural increase of the populations; the aphid populations were, however, not different from each other when the intrinsic rate of natural increase was compared and when cohort generation time was compared (Table 6 and Table 7). The interaction between host genotype and aphid population was not significant on the intrinsic rate of the natural increase of RWA populations and the cohort generation time of RWA. Genotype Kwale had the biggest effect on the intrinsic rate of natural increase compared to KRWA9 and was the best host for increasing the aphid population of RWA. Maling’a [19] reported that the aphid populations developed faster and higher populations on susceptible wheat genotypes without *Dn* genes as compared to resistant wheat genotypes, like KRWA9, that contained *Dn* genes and the progenies. This shows that resistant wheat genotypes that contain *Dn* genes can be used to manage RWA populations in Kenya.

Pearson’s correlation coefficients for aphid development time, aphid reproductive time (reproduction longevity), aphid lifespan, M_d_, total fecundity, intrinsic rate of natural increase and cohort generation time are presented in Table 8. A significant negative relationship was observed between aphid development time and M_d_ (*r* = 0.299, *p* < 0.05), indicating that populations with a shorter development time had higher progenies during the initial reproduction period. Development time was also negatively correlated with total fecundity and the intrinsic rate of natural increase. This means that the aphid population with the shortest development time has a higher intrinsic rate of natural increase and total fecundity. Cohort generation time was negatively correlated with M_d_, total fecundity and the intrinsic rate of natural increase, meaning that these parameters cannot be used to predict the cohort generation time of RWA.

Reproductive time had a significant positive correlation with aphid lifespan, M_d_, total fecundity and the intrinsic rate of natural increase. Aphid lifespan was positively correlated with M_d_, total fecundity and the intrinsic rate of natural increase, whereas M_d_ was strongly correlated with total fecundity and the intrinsic rate of natural increase. There was no correlation between development time, reproductive time, aphid lifespan and cohort generation time.

## 4. Conclusions

The studies showed that RWA collected from Eldoret, Mau Narok and Njoro differed in growth and reproductive potential, especially on susceptible host Kwale as compared to resistant KRWA9, where no differences in growth and reproductive potential were noticeable. A strong and positive correlation was found between aphid lifespan and reproductive time, total fecundity and intrinsic rate of natural increase. These are reliable predictors of aphid population growth. Significant negative correlation was found among cohort generation time, total aphid fecundity and reproductive time. Though survivorship on wheat was not good enough, a parameter to determine the existence of biotypes, other phenotypic markers used to determine biotypes were good enough to show differences among populations of RWA in Kenya. Further, the study concluded that the use of phenotypic life and reproductive markers could be used for characterizing biotypes of RWA, though this needs to be corroborated using virulence data.

## Figures and Tables

**Table 1:** Russian wheat aphid populations used in the study and their collection sites and original hosts. RWA, Russian wheat aphid; KALRO, Kenya Agricultural and Livestock Research Organization.

RWA Population	Collection Site	Location	Altitude	Original Host
Eldoret	Moi University, Chepkoilel	0.5° N, 35.3° E	3085 masl	Bread wheat
Mau Narok	Purko farm, Tipis	0.3° N, 35.9° E	2829 masl	Bread wheat
Njoro	KALRO, Njoro	0.6° S, 36.0° E	2166 masl	Bread wheat

**Table 2:** Tests between groups on different host genotypes.

Host Genotype	Test	ChiSquare	DF	Prob > ChiSq
KRWA9	Log-rank	5.5749	2	0.0616
KRWA9	Wilcoxon	3.2761	2	0.1944
Kwale	Log-rank	5.1086	2	0.0777
Kwale	Wilcoxon	5.2608	2	0.0721

**Table 3:** Effect of host genotype on development time of RWA from 1st to 4th instar development stages.

Variety	Development Time (Number of Days)				
	1st Instar	2nd Instar	3rd Instar	4th Instar	Development Time
KRWA9	4.3 ^a^	2.5 ^a^	2.1	2.0 ^b^	10.8 ^a^
Kwale	3.7 ^b^	2.1 ^b^	2.1	2.3 ^a^	9.7 ^b^
F pr	<0.001	0.002	0.3911	0.014	0.0001
SE	0.12	0.11	0.08	0.08	0.35
CV	4.6	10.0	4.6	9.2	5.6

Means not followed by the same letter are significantly different by Student’s *t*-test (α = 0.05).

**Table 4:** Effect of two wheat genotypes on the development (days) of RWA populations.

Wheat Genotype	RWA Population	Development time (Days)				
		1st Instar	2nd Instar	3rd Instar	4th Instar	Development Time
Kwale						
	Eldoret	3.5 ^c^	1.8	2.0	2.2	8.8 ^c^
	Mau Narok	4.0 ^ab^	2.4	2.2	2.2	10.4 ^ab^
	Njoro	3.6 ^bc^	2.0	2.0	2.4	10.0 ^bc^
KRWA9						
	Eldoret	4.3 ^a^	2.5	1.9	2.0	10.6 ^ab^
	Mau Narok	4.1 ^ab^	2.4	2.2	2.0	10.3 ^ab^
	Njoro	4.5 ^a^	2.5	2.2	2.1	11.5 ^a^
	F pr	0.011	0.08	0.410	0.884	0.01
	SE	0.20	0.18	0.17	0.007	0.64
	CV	12.2	15.8	19.3	9.0	14.0

Means not followed by the same letter are significantly different by Tukey’s HSD test (α = 0.05).

**Table 5:** The effect of wheat genotype on the reproduction time, total aphid lifespan, effective fecundity (M_d_) and total fecundity of RWA.

Variety	Aphid Life Parameters			
	Reproduction time (days)	Aphid lifespan (days)	M_d_	Total fecundity
KRWA9	16.9	27.93	15.60 ^b^	23.4 ^b^
Kwale	18.3	28.76	19.81 ^a^	31.2 ^a^
F pr	0.512	0.79	0.022	0.034
SE	2.1	2.4	1.7	3.4
CV	28.1	20.3	18.3	25.9

Means not followed by the same letter are significantly different by Student’s *t*-test (α = 0.05).

**Table 6:** The differences in reproduction time, total aphid lifespan, effective fecundity (M_d_) and total fecundity of RWA populations.

RWA Population	Reproduction Time (Days)	Aphid Lifespan (Days)	M_d_	Total Fecundity	Intrinsic Rate of Natural Increase	Cohort Generation Time
Eldoret	15.2 ^b^	25.47 ^b^	17.62 ^ab^	26.8 ^ab^	0.20	12.91 ^b^
Mau Narok	14.6 ^b^	25.40 ^b^	15.22 ^b^	21.2 ^b^	0.17	13.76 ^ab^
Njoro	23.1 ^a^	33.76 ^a^	20.43 ^a^	33.6 ^a^	0.21	14.30 ^a^
F pr	0.003	0.0065	0.0497	0.0266	0.0572	0.0357
SE	2.6	3.0	2.0	4.21	0.02	0.57
CV	28.1	20.3	18.3	25.9	11.6	5.6

Means not followed by the same letter are significantly different by Tukey’s HSD test (α = 0.05).

**Table 7:** Effect of host genotype (Kwale and KRWA9) on the reproduction time (days), total aphid lifespan (days), effective fecundity (M_d_) and total fecundity of populations of RWA.

RWA Population	Wheat Genotype	Reproduction Time (Days)	Aphid Lifespan (Days)	M_d_	Total Fecundity	Intrinsic Rate of Natural Increase	Cohort Generation Time
	Kwale						
Eldoret		18.4	27.7	20.1	35.6 ^ab^	0.219	14.40 ^ab^
Mau Narok		12.5	23.3	15.1	18.2 ^b^	0.163	14.40 ^ab^
Njoro		24.5	34.5	23.7	38.9 ^a^	0.234	15.19 ^a^
	KRWA9						
Eldoret		11.5	23.4	14.3	16.7 ^b^	0.179	12.59 ^c^
Mau Narok		17.4	27.5	15.8	24.7 ^ab^	0.176	14.40 ^ab^
Njoro		21.1	32.9	17.1	28.0 ^ab^	0.181	13.33 ^bc^
F pr		0.2265	0.330	0.2136	0.0401	0.1017	0.0181
SE		3.61	4.224	2.876	6.17	0.0218	0.806
CV		27.2	39.8	39.2	53.0	26.9	13.8

Means not followed by the same letter are significantly different by Tukey’s HSD test (α = 0.05).

**Table 8:** Correlation matrix for aphid development time, aphid reproductive time, aphid lifespan, effective fecundity (M_d_), total fecundity, intrinsic rate of natural increase and cohort generation time.

	Dtime	Rtime	Lifespan	M_d_	Fecundity	Rm	Tc
Dtime	1.000						
Rtime	−0.099	1.000					
Lifespan	0.007	0.994 *	1.000				
M_d_	−0.299 *	0.773 *	0.749 *	1.000			
Fecundity	−0.321 *	0.897 *	0.868 *	0.886 *	1.000		
Rm	−0.529 *	0.673 *	0.624 *	0.921 *	0.800 *	1.000	
Tc	1.000	−0.098	0.008	−0.298 *	−0.321 *	−0.529 *	1.000

*r*_(0.05,64)_ = 0.250; * significant correlation at *p* = 0.05.
